# HIV-free survival among nine- to 24-month-old children born to HIV-positive mothers in the Rwandan national PMTCT programme: a community-based household survey

**DOI:** 10.1186/1758-2652-15-4

**Published:** 2012-01-30

**Authors:** Hinda Ruton, Placidie Mugwaneza, Nadine Shema, Alexandre Lyambabaje, Jean de Dieu Bizimana, Landry Tsague, Elevanie Nyankesha, Claire M Wagner, Vincent Mutabazi, Jean Pierre Nyemazi, Sabin Nsanzimana, Corine Karema, Agnes Binagwaho

**Affiliations:** 1 Rwanda Biomedical Center, Kigali, Rwanda; 2 School of Public Health, National University of Rwanda, Kigali, Rwanda; 3 UNICEF, Kigali, Rwanda; 4 Department of Global Health and Social Medicine, Harvard Medical School, Boston, USA; 5 Ministry of Health, Kigali, Rwanda

**Keywords:** children and HIV, vertical transmission of HIV, PMTCT, programme effectiveness, elimination of MTCT, HIV-free survival, Africa, Rwanda

## Abstract

**Background:**

Operational effectiveness of large-scale national programmes for the prevention of mother to child transmission (PMTCT) of HIV in sub-Saharan Africa remains limited. We report on HIV-free survival among nine- to 24-month-old children born to HIV-positive mothers in the national PMTCT programme in Rwanda.

**Methods:**

We conducted a national representative household survey between February and May 2009. Participants were mothers who had attended antenatal care at least once during their most recent pregnancy, and whose children were aged nine to 24 months. A two-stage stratified (geographic location of PMTCT site, maternal HIV status during pregnancy) cluster sampling was used to select mother-infant pairs to be interviewed during household visits. Alive children born from HIV-positive mothers (HIV-exposed children) were tested for HIV according to routine HIV testing protocol. We calculated HIV-free survival at nine to 24 months. We subsequently determined factors associated with mother to child transmission of HIV, child death and HIV-free survival using logistic regression.

**Results:**

Out of 1448 HIV-exposed children surveyed, 44 (3.0%) were reported dead by nine months of age. Of the 1340 children alive, 53 (4.0%) tested HIV positive. HIV-free survival was estimated at 91.9% (95% confidence interval: 90.4-93.3%) at nine to 24 months. Adjusting for maternal, child and health system factors, being a member of an association of people living with HIV (adjusted odds ratio: 0.7, 95% CI: 0.1-0.995) improved by 30% HIV-free survival among children, whereas the maternal use of a highly active antiretroviral therapy (HAART) regimen for PMTCT (aOR: 0.6, 95% CI: 0.3-1.07) had a borderline effect.

**Conclusions:**

HIV-free survival among HIV-exposed children aged nine to 24 months is estimated at 91.9% in Rwanda. The national PMTCT programme could achieve greater impact on child survival by ensuring access to HAART for all HIV-positive pregnant women in need, improving the quality of the programme in rural areas, and strengthening linkages with community-based support systems, including associations of people living with HIV.

## Background

Worldwide, 2.5 million children younger than 15 years were living with HIV in 2009, and more than 90% had been infected via mother to child transmission (MTCT) of HIV during pregnancy, delivery and breastfeeding [1]. Developed countries have achieved remarkable progress in the prevention of MTCT (PMTCT) by scaling up access to highly active antiretroviral therapy (HAART), elective caesarean section, and formula feeding as a replacement to breastfeeding, leading to a dramatic decrease in the MTCT rate (below 2%) [2-4]. Yet MTCT remains a major challenge in developing countries, particularly in sub-Saharan Africa where more than 90% of new paediatric HIV infections occur each year [5-7].

Recent years have nonetheless seen remarkable increases in the coverage of PMTCT programmes in sub-Saharan Africa, with a growing number of countries expanding access to comprehensive and more efficacious PMTCT interventions. By December 2009, 22 countries in sub-Saharan Africa had reported antiretroviral (ARV) coverage rates of more than 50% for all HIV-positive pregnant women, including 15 countries passing the universal access target of 80% [8,9]. There is renewed global commitment and increased financial support to achieve the goal of elimination of vertical transmission of HIV (an MTCT rate of less than 5% among all pregnant women living with HIV and a 90% reduction in new paediatric HIV infections), with improved maternal, newborn and child survival by 2015 [10]. The 2010 World Health Organization's (WHO's) revised guidelines for the use of ARV in pregnancy and breastfeeding have provided an additional opportunity for sub-Saharan Africa countries to significantly avert new HIV infections in infants while improving maternal and child health and survival [11].

In 2010, WHO and the Inter Agency Task Team on Prevention of HIV Infection in Pregnant Women, Mothers and their Children outlined the strategic vision, goals and global framework for countries to achieve elimination of MTCT by 2015 [12,13]. Measuring the effectiveness of national PMTCT programmes is a cornerstone of this strategy. HIV-free survival was proposed as the gold standard for measurement of PMTCT programme effectiveness, as it captures direct effects (such as HIV infection at birth and through breastfeeding, deaths prevented and indirect benefits of PMTCT programmes) and indirect effects (survival benefits that may accrue to HIV-exposed children who do not become infected) of PMTCT [7]. Yet there is a paucity of data on HIV-free survival rates measured at the level of the national PMTCT programme. The effectiveness of PMTCT depends not only on the efficacy of the ARV interventions, but also on client- and health system-related determinants of access and utilization of maternal and child health interventions, such as of antenatal care (ANC), facility-based delivery and post-natal care [14-16].

Rwanda is located in eastern Africa, with more than 10 million inhabitants and a prevalence of HIV estimated at 3% in the general population and 4.3% among pregnant women. The Rwanda demographic and health survey conducted in 2010 shows that 98% of all Rwandan women who had live births between 2005 and 2010 attended at least one antenatal care visit.

An estimated 200,000 people are living with HIV, among them 22,000 children younger than 15 years [17]. The government of Rwanda aims at elimination of MTCT as outlined in its 2008-2012 National Strategic Plan for HIV&AIDS (NSP) response. The national PMTCT programme has been established since 2001 by the Ministry of Health through the Center for Treatment and Research on AIDS, Tuberculosis, Malaria and Other Epidemics (TRAC *Plus*), and 98% of all Rwandan women who attended PMTCT services between 2006 and June 2010 accepted testing for HIV (Ministry of Heath, TRAC Plus). However, its effectiveness has not yet been assessed. Recent studies have reported HIV-free survival of 94-95% by nine months of age in a clinical trial [18,19] and 95% by 12 to 18 months in district-level PMTCT programme [20] in Rwanda. The present study was initiated in 2009 to assess the effectiveness of the Rwanda national PMTCT programme through the measurement of nine- to 24-month HIV-free survival and its determinants. This will serve as a baseline for monitoring national trends toward achievements of the NSP targets by 2012.

## Methods

### PMTCT programme description

After a successful pilot phase (1999) in one site (Kicukiro health centre in Kigali), Rwanda established the national PMTCT programme in 2001, coordinated by the Treatment of Research on AIDS Center (TRAC *Plus*) under the Ministry of Health. In 2006, Rwanda developed and costed a national PMTCT scale-up plan (2007-2012) with population-based targets that articulated a comprehensive strategy to deliver a comprehensive package of PMTCT interventions integrated within MCH services at the community level. PMTCT programme scale up and coordination is supported by various partners, including bilateral (Global Fund and PEPFAR), multilateral (United Nations' One UN Programme) and civil society organizations. Routine provision of PMTCT services was effective in 75% of health facilities in Rwanda by 2009 (up from 45% in 2005) [21], and increasing number of male partners of pregnant women were being tested for HIV (from 33% in 2005 to 78% in 2009). Rwanda promotes the integration of PMTCT services within routine maternal and child health care, including family planning services.

**Figure 1:**
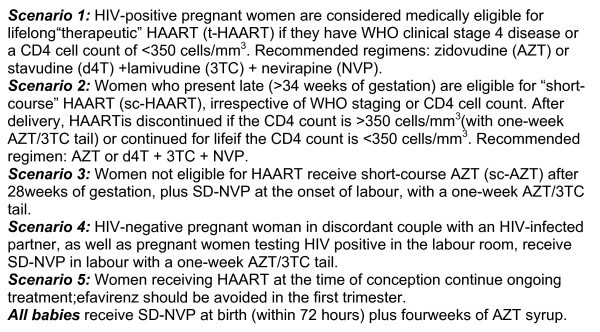
**Scenarios**.

In 2005, Rwanda transitioned from single-dose nevirapine (sd-NVP) to more efficacious ARV regimens for PMTCT and rolled this out to all PMTCT sites by 2008 [22-25] (See Figure 1). Routine CD4 count screening during pregnancy and services for early infant diagnosis of HIV using dried blood spot tests were introduced. A national task-shifting policy was instituted in 2009 to support the effective initiation of HAART by nurses at the decentralized level. By 2009, 61% of HIV-positive pregnant women received ARV for PMTCT [26], and about 92% of them received more efficacious ARV regimens [26].

In November 2010, Rwanda adopted option B of the recent WHO recommendations for use of ARVs in PMTCT. This adoption permits all HIV-positive pregnant women to receive HAART, starting at different stage of their pregnancy. Mothers who do not meet the WHO for Affordable Feasible Accessible Safe and Sustainable formula feeding (AFASS) criteria continue ARVs throughout the entire breastfeeding period [11].

### Study design

#### Study population and sampling methodology

This community-based household survey was conducted between February and May 2009 and targeted mothers (regardless of HIV status) who had previously attended the PMTCT programme during their most recent pregnancy (at least one ANC visit), and their nine- to 24-month-old children (born between March 2007 and June 2008). A stratified two-stage cluster sampling process was used to select participants. The first stage included selection of a sample of PMTCT sites from a stratified (urban vs. rural) sample of sites that had offered PMTCT services for at least 36 months prior to the survey. The second stage was done at the site level using ANC registers; mothers across three strata were selected: HIV-positive mothers who completed at least four ANC visits; HIV-positive mothers who completed less than four ANC visits; and HIV-negative mothers. Assuming an MTCT rate of 35% and 25%, respectively, without and with PMTCT programme available, a significance level of 5%, a power of 80%, a non-response rate of 10% and a design effect of 2, we estimated that 3420 mother-child pairs were required for the study's sample size. For this paper, we will focus on the HIV-positive mothers.

Mother-child pairs identified in the clinic roster that could not be found at the location given as their home place were replaced. Researchers returned to the health facility and selected other mother-child pairs to complete the total sample size. Very few mother-child pairs could not be located. The replacement of missing pairs did not cause underestimation of child mortality as the only reason they could not be found was because the address had been incorrectly documented or the pair had moved.

#### Data collection

Household visits were conducted by a team composed of one counsellor from the selected health facility, one data collector and one laboratory technician. A standardized and validated questionnaire was used to collect data during interviews conducted in Kinyarwanda, the national language of Rwanda. Each interview lasted on average for 45 minutes. The questionnaire included maternal knowledge of HIV in general and PMTCT in particular, the use of PMTCT services by HIV-positive women, and the use of reproductive health services. Child demographics, anthropometric data and HIV status were recorded in a separate tool.

At the time of the interview, the mother was asked if the child was alive or deceased. If the child was deceased, the mother was asked for the exact date when her child had died. The time between birth and death was recorded and thus was available for analysis. HIV testing was done on the day of the interview. If the child had died, the household visit stopped after the questionnaire. The variable in follow-up time derives from the computation of the time between birth and death (if the child had died) or birth and interview date (if the child was alive).

For children who did not survive, HIV infection status was impossible to determine. If the child was alive, and after obtaining informed verbal consent from parents, a blood sample was collected from all HIV-exposed children aged nine to 24 months using heel or finger prick. We performed rapid HIV testing on the spot for all children according to national HIV testing algorithm on three parallel rapid tests: Determine (Abbott Laboratories, Abbott Park, IL) as the first test, Unigold (Trinity Biotech) as confirmatory, and Capillus as a tie-breaker in cases of discordancy between first and second test. Polymerase chain reaction (PCR) was used to confirm all HIV-positive rapid tests among children younger than 18 months, and for indeterminate or discordant results between the three rapid test results. In all other cases, results of the three rapid tests were considered as final. For quality control, every HIV rapid test batch was checked for optimal performance using negative and positive controls by the laboratory technician in the field before the testing of the samples collected. One out of every 20 samples tested with HIV rapid tests was collected on a dried blood spot card and retested with PCR. All PCR tests were performed at the National Reference Laboratory in Kigali.

#### Ethical considerations

The study was approved by the National Institute of Statistics of Rwanda and the Rwanda National Ethics Committee. Appropriate measures were taken to ensure survey participant protection, and informed consent of mothers was secured prior to interview. Participation was voluntary and patient confidentiality was ensured.

#### Data management and analysis

Completed questionnaires were periodically brought in from the study sites to the School of Public Health of the National University of Rwanda for data entry and analysis. Data were entered using the Census and Survey Processing System (CSPro 4.0). A quality control programme was used to detect data collection and/or data entry errors. This information was shared with field teams during supervisory visits and weekly meetings to improve data quality. In addition, 10% of the questionnaires were double entered for data quality control at the initiation of data entry. Data was exported to STATA 10.1 for data analysis. We focused only on data from HIV-positive mothers and their HIV-exposed children for this analysis.

#### Study endpoint

The primary study endpoint was HIV-free survival at nine to 24 months. Survival was defined as the probability that the child was alive between nine and 24 months of age, and tested HIV negative at this age.

#### Predictor variables

The potential predictor variables were divided into three categories: individual, household variables and measures of PMTCT of HIV interventions. Individual and household variables included socio-demographic characteristics of mother and child, decision-making power about health and nutrition index, decision-making power about short- and long-term investment strategies index, housing material index, household asset index, household size, access to clean water, and geographic location of household. The measure of PMTCT interventions included the type of ARV taken by mother and infant, place of delivery, and infant feeding (whether or not the infant was appropriately fed through the time of the survey). Appropriate feeding options for HIV-exposed children included at the time of the survey were: exclusive breastfeeding from birth with early cessation of all forms of breastfeeding by four to six months (early weaning); or exclusive replacement from birth if AFASS criteria are met.

### Statistical analysis

We determined HIV-free survival as one minus the combined risk of HIV infection and/or death. All confidence intervals (CI) were at the 95% level of significance, and p values of 0.05 were considered statistically significant. We then modelled the risk of HIV infection by nine to 24 months and/or dying by the age of nine months among children born to HIV-positive mothers by logistic regression models. Stepwise selection with a probability of 0.05 for variables to enter the model and a probability of 0.15 to be removed from the model was used to identify determinants of either acquiring HIV infection and/or dying among HIV-exposed children.

## Results

### Description of survey participants

**Figure 2:**
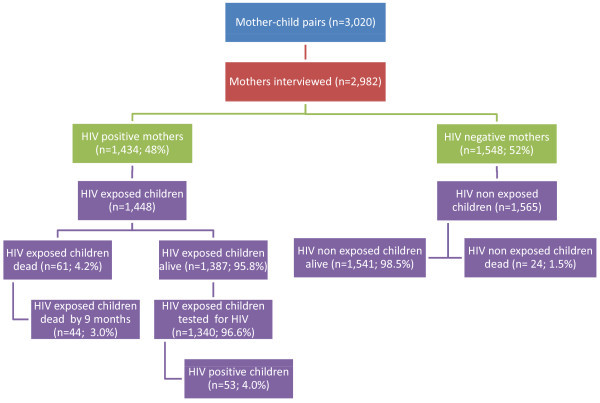
**Study overview**.

The study was conducted in 105 health facilities in five provinces. On average, 13.9 HIV-positive mother-child pairs were interviewed per health facility, with a standard deviation of 8.2. We interviewed a total of 1434 HIV-positive mothers and their 1455 HIV-exposed children (Figure 2). The mean age of HIV-positive mothers interviewed was 32.2 years. The majority of women lived in rural areas (80%), had a partner (69%), were aged between 25 and 39 years (78%), and had a primary school education level (68%) (Table 1). The majority of children stopped exclusive breastfeeding at six months (84%) and 91% at nine months, and were aged between 13 and 24 months (74%). (Table 2).

**Table 1:** Background characteristics of HIV-positive mothers surveyed, Rwanda PMTCT programme, 2009

Characteristics		Number	%
**Mother's age groups (years), (n = 1430)**	15-24	142	9.9
	25-29	354	24.8
	30-34	415	29.0
	35-39	351	24.6
	40-49	168	11.8
**Mean age (years)**,		32.2	
**Marital status (n = 1429)**	Single/never married	122	8.5
	Lives with a partner	986	69.0
	Separated/divorced/widowed	321	22.5
**Religion (n = 1432)**	No religion	23	1.5
	Adventist	141	9.9
	Catholic	609	42.5
	Protestant	582	40.6
	Muslim	60	4.2
	Others	17	1.2
**Education (n = 1428)**	Never attended school	353	24.7
	Primary school	965	67.6
	Vocational/technical	37	2.6
	Secondary school	69	4.8
	University	4	0.3
**Literacy (n = 1431)**	can't or have difficult reading and/or writing	573	40.04
	can read but can't or have difficult writing	69	4.82
	can read and write easily	789	55.14
**Residence (n = 1434)**	Rural	1141	79.6
	Urban	293	20.4
**Membership to a PLHIV association (n = 1417)**	Yes	775	54.7
	No	642	45.3
**ARV and/or cotrimoxazole received by mother (n = 1383)**	None	377	28.3
	Cotrimoxazole	308	23.1
	NVP alone	156	11.7
	Dual therapy	108	8.1
	Triple therapy	384	28.8

**Table 2:** Background characteristics of children born from HIV-positive mother, Rwanda PMTCT programme, 2009

Characteristics		Number	%
**Child's age (months) (n = 1357)**	< = 12	350	25.8
	13-17	474	34.9
	> = 18	533	39.3
**Child's sex (n = 1348)**	Female	719	49.65
	Male	729	59.35
**Feeding (n = 1360)**	Appropriate	117	8.6
	Inappropriate	1,243	91.4
**Month of breastfeeding (n = 1161)**	< = 6	973	83.8
	7-9	84	7.2
	10-12	41	3.5
	> 12	63	5.4
**Child received ARV at birth (n = 1394)**	None	79	5.7
	Nevirapine	536	38.5
	Dual therapy	482	34.6
	Don't know	297	21.3

### Distribution of ARV regimens for PMTCT among HIV-positive mothers

Of the 1434 HIV-positive women interviewed, information on the use of ARV or co-trimoxazole during pregnancy for PMTCT was not available for 101 (7.0%). Of the 1333 (93.0%) women who reported the use of any ARV or co-trimoxazole for PMTCT during pregnancy (Table 1), 956 (72%) had received some ARV while 377 (28%) had not. ARV or co-trimoxazole received for PMTCT included HAART (29%), dual ARV prophylaxis (8%), sd-NVP (12%) and co-trimoxazole (21%).

### HIV infection among children alive at age 9-24 months and associated factors

Of the 1340 children alive aged nine to 24 months and tested for HIV, 53 were positive, with an estimated HIV prevalence of 4.0% (95% CI: 2.9-5.0). Maternal age was the only factor associated with HIV infection among children, with the highest HIV prevalence (6.8%) among children born to young mothers (15 to 24 years) and the lowest among children born to women aged 35 to 39 years (adjusted odds ratio [aOR]: 0.3, 95% CI: 0.1-0.9) (Tables 3 and 4). In multivariate analysis, children born to HIV-positive women who received HAART during pregnancy were 60% less likely to be infected with HIV when compared with children whose mothers did not take any ARV during pregnancy (aOR: 0.4, 95% CI: 0.1-0.96) (Table 3).

**Table 3:** Prevalence of HIV infection among HIV-exposed children by PMTCT programme indicators and maternal demographic characteristics, Rwanda PMTCT programme, 2009

Characteristics	HIV-positive children (%)	Univariate analysis (*p*)	Multivariate analysis	
			**OR**	**95%CI**
			
**Child received ARV at birth (n = 1340)**		0.3		
None (reference)	6.5		1	
NVP	4.5		0.7	0.2-2.2
Dual therapy	3.6		0.5	0.2-2.1
Don't know	2.9		0.4	0.1-1.9
**Mother delivered at health centre (n = 1336)**		0.4		
Yes	3.8		0.9	0.3-3.0
No (reference)	5.2		1	
**Feeding options at birth (n = 1303)**		0.1		
EBF (reference)	3.4		1	
BF and early cessation	4.0		0.9	0.4-1.7
Formula	2.3		0.7	0.2-2.4
Animal modified milk	1.2		0.3	0.0-2.5
				
**Number of ANC visits (n = 1340)**		0.4		
Less than four (reference)	4.3		1	
Four or more	3.5		1.2	0.6-2.1
**ARV and/or cotrimoxazole taken by the mother (n = 1333)**		0.05		
None (reference)	5.4		1	
Cotrimoxazole	3.1		0.6	0.3-1.4
NVP alone	7.0		1.2	0.5-3.0
Dual therapy	4.8		1.1	0.4-3.0
Triple therapy	1.9		0.4	0.1-0.96*
				
**Mother's age (years) (n = 1337)**		0.01*		
15-24 (reference)	6.8		1	
25-29	4.8		0.9	0.4-2.3
30-34	4.7		0.8	0.3-1.9
35-39	1.5		0.3	0.1-0.9
40-49	3.0		0.6	0.2-2.0

* Significant (p < 0.05 for univariate analysis); OR does not include 1 for multivariate analysis.

**Table 4:** Cumulative risk of HIV infection by 9-24 months and/or death by 9 months old among HIV-exposed children, Rwanda PMTCT programme, 2009

**Child's outcome**	**Total number of children assessed**^**&**^	**No % (95% CI)**	**Yes % (95% CI)**
	
Death	1455	95.8 (94.8-96.9)	4.2 (2.1-3.9)
HIV infection	1340	96.0 (95.0-.97.1)	4.0 (2.9-5.0)
HIV infection and/or death	1401	91.9* (90.4-.93.3)	8.1 (6.7-9.6)

^&^The total number of children assessed represents the population of HIV-exposed children assessed for each outcome

* The value in this cell estimates the proportion of children alive and free of HIV, defined as HIV-free survival by 9-24 months

### HIV-free survival by 9-24 months and associated factors

Of 1455 children born to HIV-positive mothers, 61 (4.2%) were reported dead, including 44 (3.0%) by nine months (Figure 2). Among 1340 children aged nine to 24 months who were alive and tested for HIV, 53 (4.0%) tested HIV positive, translating into a nine- to 24-month HIV-free survival rate of 91.9% (95% CI: 90.4-93.3%) (Table 4). Children whose mothers were members of a people living with HIV (PLHIV) association (aOR: 0.7, 95% CI: 0.4-1.0.8) were 30% (aOR: 0.7, 95% CI: 0.3-0.995) less likely to be infected with HIV and/or dead compared with children whose mothers did not receive any ARV during pregnancy (Table 5). Also, children whose mothers received HAART for PMTCT were (aOR: 0.6, 95% CI: 0.3-1.07) 40% less likely to have HIV infection than living children whose mothers had not received HAART (Table 6).

**Table 5:** Factors associated with HIV-free survival among HIV-exposed infants, Rwanda PMTCT programme, 2009

	HIV-free survival	
	**Adjusted odds ratio**	**95% CI**
	
ARV and/or cotrimoxazole taken by the mother		
None	Reference	1
Cotrimoxazole	0.9	0.5-1.5
NVP alone	1.5	0.8-2.7
Dual therapy	0.6	0.3-1.5
Triple therapy	0.6	0.3-1.07
Location		
Rural	Reference	1
Urban	0.7	0.3-1.2
Membership of a PLHIV association, (n = 1417)		
No	Reference	1
Yes	0.7	0.4-0.995

**Table 6:** Prevalence of HIV infection among HIV exposed children according to socio-demographic characteristics (mothers and children), Rwanda PMTCT programme, 2009

Characteristics	HIV-positive children	HIV-negative children	Univariate analysis (p)	Multivariate analysis	
				**OR**	**95%CI**
				
**Mothers characteristics**					
**Mother's age (years), % n = (1337)**			0.01*		
15-24 (reference)	6.8	93.2		1	
25-29	4.8	95.2		0.7	0.3-1.8
30-34	4.7	95.3		0.6	0.2-1.5
35-39	1.5	98.5		0.2	0.1-0.8
40-49	3.1	96.9		0.4	0.1-1.4
**Marital status,% n = (1336)**			0.76		
Single/never married (reference)	6.1	93.9		1	
Married/cohabiting	3.4	96.6		0.7	0.3-2.1
Separated/divorced/widowed	4.3	95.7		1.3	0.4-4.2
**Literacy,% n = 1338)**			0.77		
can't or have difficult reading and/or writing (reference)	3.7	96.3		1	
can read but can't or have difficult writing	4.6	95.4		1.2	0.4-4.6
can read and write easily	4.1	95.9		0.9	0.5-1.8
**Decision-making power in health and nutrition issues index^#^, mean (SE) (n = 1336)**	89.4(2.8)	90.2 (0.5)	0.78	1.0	0.98-1.02
**Decision-making power in short- and long-term investment strategies index^#^, mean (SE) (n = 1336)**	82.1(3.8)	84.3(07)	0.54	0.9	0.98-1.01
**Membership to a PLHIVassociation, % (n = 1323)**			0.13		
Yes	3.3	96.7		0.6	0.3-1.1
No (reference)	4.9	95.1		1	
**Household characteristics**					
**Household size, mean (SE) (n = 1311)**	5.0(0.36)	5.1(0.05)	0.57	1.0	0.8-1.1
**Housing index^@^, mean (SE) (n = 1313)**	1.5(0.12)	1.4(0.02)	0.14	1.4	0.9-2.0
**Household assets index^$^, mean (SE) (n = 1329)**	1.0(0.13)	0.9(0.03)	0.71	1.2	0.9-1.6
**Access to clean water, % (n = 1323)**			0.40		
Yes	4.2	95.8		1.6	0.6-4.2
No (reference)	3.0	97.0		1	
**Location, % (n = 1340)**			0.19		
Rural	4.3	95.7		1	
Urban	2.55	97.45		0.3	0.2-1.05
**Children characteristics**					
**Child's sex (n = 1338)**			0.89		
Female (reference)	4.0	96.0		1	
Male	3.9	96.1		0.8	0.5-1.7
**Child's age (months),% (n = 1340)**			0.23		
< = 12	3.8	96.2		1	
13-17	2.8	97.2		0.6	0.3-1.4
> 18	5.1	94.9		1.2	0.6-2.5

* Significant p < 0.05

**^# ^**Decision-making power about health and nutrition index is defined as a composite index created to measure the extent to which the respondent is involved in making household decisions related to health and nutrition issues. Scores ranged from 0 to 100 and a higher score indicates greater decision-making power. Decision-making power about short- and long-term investment strategies index is defined as a composite index created to measure the extent to which the respondent is involved in making household decisions related to short and long-term investment issues.

^@^Housing material index is defined as a composite index indicating whether roof, walls, and floors of the respondent's house are built in durable materials. The index ranged from 0 to 3: 0 means that none of the housing materials are durable while 3 means that materials for roof, walls and floors are durable.

^$ ^Household asset index is defined as a composite index indicating the number of valuable equipments owned (refrigerators, mobile telephone, radio, TV, car, etc.).

## Discussion

This is, to our knowledge, the first evaluation of HIV-free child survival in a national PMTCT programme in a resource-limited setting. We reported a rate of HIV-free survival of 91.9% among children born to HIV-positive mothers in the Rwanda national PMTCT programme by nine to 24 months of age. This figure is higher than the one reported at nine months (65-84%) in an effectiveness evaluation of the PMTCT programme that was limited to three districts in South Africa [27]. However, the comparison between the two evaluations is limited in that the South African evaluation was conducted five years prior to the one in Rwanda, at a time when single-dose nevirapine was the only regimen used in South Africa.

Nevertheless, the nine- to 24-month HIV-free survival rate in the Rwanda national PMTCT programme remains comparable to the nine-month HIV-free survival rate of 95% (91-97%) reported in a clinical trial conducted in Rwanda in 2005-2007 comparing six months of HAART during breastfeeding with formula feeding [19]. Although the majority of women (83.3%) in our study reported breastfeeding their child from birth compared with 10.1% who provided formula, our HIV-free survival rate at nine to 24 months is similar to the 12- to 18-month HIV-free survival rate of 95% (91-97%) reported in a recent evaluation of a district level PMTCT demonstration programme providing a comprehensive package of health services, including free formula from birth for all HIV-exposed infants [28]. Yet, the national PMTCT programme in Rwanda does not support provision of free formula for all HIV-exposed children, instead promoting the implementation of the WHO AFASS criteria [29] to determine the best feeding option for each HIV-exposed infant. Our findings show that infant feeding options at birth have an impact on HIV-positive status; however, we do not have enough statistical power to detect a difference.

The World Health Organization has reported that HIV-free survival is related to the HIV MTCT rate and survival regardless of HIV infection in a cohort of HIV-exposed children [7]. It is a dynamic parameter that decreases with time depending on the cumulative risk of transmission (during exposure through breastfeeding), access to HIV preventive methods during breastfeeding, and access to the basic child survival package of interventions. Based on the HIV prevalence among HIV-exposed children (3.0%) and assuming that all reported child deaths were HIV-positive cases (worst-case scenario), the cumulative HIV transmission rate in the Rwanda national PMTCT programme is estimated at 8.1% by nine to 24 months. This is likely to be an overestimation of the true HIV transmission rate, given that exposure to HIV has been recently reported to contribute to only 3.6% to 4% of under-five mortality in sub-Saharan Africa [30,31]. Nevertheless, this is comparable with the cumulative HIV transmission rate of 5.7% (95% CI: 2.5-9.0%) at 12 months from the MTCT-Plus Initiative in Abidjan, Cote d'Ivoire, providing HAART or short-course ARV to HIV-positive pregnant women for PMTCT [32].

Our study showed a 40% reduction in likelihood of death or HIV infection by nine to 24 months when mothers initiated HAART for PMTCT, consistent with previous reports of increased HIV-free child survival with use of maternal HAART initiated during pregnancy and extended during breastfeeding [19,33,34]. Breastfeeding with early cessation was reported by 33% of HIV-positive mothers; however, mode of breastfeeding was not associated with HIV-free survival in our study, although previous randomized trials have provided evidence of the negative impact of early and abrupt cessation of breastfeeding on HIV-free survival of children [35,36]. The Rwandan Ministry of Health has already set new national standards and guidelines for PMTCT in line with the 2010 WHO guidelines [11], choosing the option B regimen (every HIV-positive pregnant woman will receive HAART as a lifelong treatment if eligible or as prophylaxis until one week after cessation of breastfeeding) [37].

We reported in our study that 63% of HIV-positive mothers had received any ARV during pregnancy, indicating that more than one-third of women did not receive ARV. More efforts will be required to increase coverage and access to ARVs for mothers and children as Rwanda transitions to option B [38] to improve both maternal health and child survival. Other factors likely to reduce barriers to access and utilization of these services in Rwanda include: the community-based health insurance covering 91% of households by 2010; the well-functioning community health programme; and a national system of performance-based financing for health services [39].

Our study showed that psychological and social support systems organized for PLHIV were beneficial, reducing by 30% the risk of death or HIV infection by nine to 24 months among HIV-exposed children. Many studies have found a positive association between social support and better health outcomes [40-42]. In a study conducted among people living with HIV in the United States of America, social support was found to be a robust predictor of health outcomes over time, independent of coping style and baseline medical status [43]. In addition, the involvement of men in PMTCT has significantly increased over the past five years in Rwanda to reach 84% in 2009 (up from 33% in 2005), and has been reported to significantly contribute to improved HIV-free survival by 12 months [44].

Our study bears some limitations. We used a cross-sectional design, and were not able to determine HIV status of infants who had died prior to the survey. In addition, recall bias may have occurred as mothers were asked to report on events that had happened nine to 24 months earlier, including ARV treatment and HAART regimens.

Another limitation of the study is the wide time interval considered (nine to 24 months). Survival rates for children who were one month old are different than those at 24 months old; thus the timespan may cause an overestimation or underestimation of survival rates. Further, some of the children were still being breastfed at the time of the interview. As such, these children were still susceptible to infection and thus our probability may be slightly underestimated.

A final limitation concerns the small number of mother-child pairs who could not be found according to the addresses given in the clinic rosters. Since all mother-child pairs were replaced immediately if they could not be located, our data do not include the number of pairs who were not included in the analysis due to an inability to find them. Rwanda is launching a six-week effectiveness study of the national PMTCT programme in 2011 that will provide insights into early HIV transmission rate, and estimate its association with various factors that were not assessed in our study, including maternal CD4 cell count.

## Conclusions

Measuring the effectiveness of the national PMTCT programme is imperative to determining the best course of action at the country level, and to making better use of the limited financial resources available. Rwanda is leading the way and has reported a rate of HIV-free survival of 91.9% at nine to 24 months of age among HIV-exposed children, a rate that can be further improved by increasing access to HAART among HIV-positive pregnant women and by ensuring that women receive psychological and social support from community-based systems. As the world is gearing towards achieving the goal of elimination of MTCT by 2015 [10], it is a strategic imperative for national PMTCT programmes in sub-Saharan Africa to assess the effectiveness of their interventions

## Competing interests

The authors declare that they have no competing interests.

## Authors' contributions

HR, PM, NS, AL, JDB, LT, EN, VM, JPN and SN contributed to the design of the study. HR, PM, NS, AL, JDB, LT, EN, VM and JPN contributed to conducting the survey, data collection and management. HR, PM, NS, AL, JDB, LT, EN, VM, JPN, SN, CK and AB contributed to the data analysis, interpretation and the concept of this manuscript. HR, PM, NS, AL, JDB, LT, EN, CMW, VM, JPN, SN, CK and AB wrote the manuscript and provided substantial intellectual content. All the authors reviewed the final manuscript.

## Authors' information

Hinda Ruton is head of the biostatistics unit at the HIV, AIDS and STIs Unit of the Center for Treatment and Research on AIDS, Malaria, Tuberculosis and Other Epidemics, Kigali, Rwanda.
